# The cause of urinary symptoms among Human T Lymphotropic Virus Type I (HLTV-I) infected patients: a cross sectional study

**DOI:** 10.1186/1471-2334-7-15

**Published:** 2007-03-12

**Authors:** Paulo N Rocha, Ana Paula Rehem, Juliana F Santana, Neviton Castro, Andre L Muniz, Katia Salgado, Heonir Rocha, Edgar M Carvalho

**Affiliations:** 1 Immunology Service, University Hospital Professor Edgard Santos, Federal University of Bahia, Rua Joao das Botas, s/n, 40110-160 Salvador, BA, Brazil

## Abstract

**Background:**

HTLV-I infected patients often complain of urinary symptomatology. Epidemiological studies have suggested that these individuals have a higher prevalence and incidence of urinary tract infection (UTI) than seronegative controls. However, the diagnosis of UTI in these studies relied only on patient information and did not require confirmation by urine culture. The purpose of this study was to investigate the role of urinary tract infection (UTI) as the cause of urinary symptoms in HTLV-I infected patients.

**Methods:**

In this cross sectional study we interviewed, and cultured urine from, 157 HTLV-I seropositive individuals followed regularly at a specialized clinic. All patients were evaluated by a neurologist and classified according to the Expanded Disability Status Scale (EDSS). Urodynamic studies were performed at the discretion of the treating physician.

**Results:**

Sixty-four patients complained of at least one active urinary symptom but UTI was confirmed by a positive urine culture in only 12 of these patients (19%); the majority of symptomatic patients (81%) had negative urine cultures. To investigate the mechanism behind the urinary complaints in symptomatic individuals with negative urine cultures, we reviewed the results of urodynamic studies performed in 21 of these patients. Most of them (90.5%) had abnormal findings. The predominant abnormalities were detrusor sphincter hyperreflexia and dyssynergia, findings consistent with HTLV-I-induced neurogenic bladder. On a multivariate logistic regression, an abnormal EDSS score was the strongest predictor of urinary symptomatology (OR 9.87, 95% CI 3.465 to 28.116, P < 0.0001).

**Conclusion:**

Urinary symptomatology suggestive of UTI is highly prevalent among HTLV-I seropositive individuals but true UTI is responsible for the minority of cases. We posit that the main cause of urinary symptoms in this population is neurogenic bladder. Our data imply that HLTV-I infected patients with urinary symptomatology should not be empirically treated for UTI but rather undergo urine culture; if a UTI is excluded, further investigation with urodynamic studies should be considered.

## Background

Human T Cell Lymphotropic Virus type I (HTLV-I) is a retrovirus that infects T lymphocytes and may cause a wide spectrum of clinical manifestations, including HTLV-I associated myelopathy/tropical spastic paraparesis (HAM/TSP), uveitis, and adult T cell leukemia. Fortunately, the majority of seropositive individuals have no signs or symptoms of illness attributable to HTLV-I infection [1]. However, a series of epidemiological studies have suggested that HTLV-I-infected individuals are at increased risk for urinary tract infections (UTI) [1-3].

The initial report of an association between HTLV-I seropositivity and UTI was published in 1997 by the Retrovirus Epidemiology Donor Study (REDS) group[1]. In this report, asymptomatic adult blood donors recruited from 11 United States centers were asked at study entry if they had been diagnosed with bladder or kidney infection within the last 5 years. This cross-sectional data revealed a higher prevalence of UTI among HTLV-I seropositive individuals compared to seronegative controls (24% versus 14.9%, P < 0.01). However, when adjusted for important covariates in a multivariate model, the association between HTLV-I seropositivity and UTI lost statistical significance. In a subsequent study, the authors measured the occurrence of UTI in the REDS cohort over a median of two years [2]. HTLV-I-infected subjects were significantly more likely to report bladder or kidney infection than seronegative controls (17.6% versus 7.8%); in this study, the association remained statistically significant even after multivariable adjustment for relevant confounding variables (OR 2.79, 95% CI 1.63 to 4.79, P < 0.05). A more recent analysis of the same cohort with longer follow-up arrived at similar findings [3].

It should be noted that in none of the above-mentioned studies urine cultures were required to define UTI; the investigators relied solely on patients' recollection of an episode of UTI that was diagnosed and treated by a physician [1-3]. This may have led to a recall bias, as seropositive individuals are more likely to recall intercurrent illnesses than healthy controls. Additionally, it is conceivable that urinary symptoms related to neurogenic bladder due to incipient HAM/TSP may have been misinterpreted (and empirically treated) as infection, leading to an over diagnosis of UTI in the seropositive group. To further address the association between urinary symptoms and UTI in this population, we prospectively interviewed and performed urine culture on 157 HTLV-I-infected individuals followed regularly at an ambulatory clinic located at a tertiary, university-based medical center in Salvador, Bahia, Brazil.

## Methods

### Patients

All patients were recruited from the HTLV-I ambulatory clinic at Hospital Universitário Professor Edgard Santos (HUPES), in Salvador, Bahia, which is the Brazilian city with the highest prevalence of HTLV-I seropositivity in the country [4]. This clinic is a referral center for local blood banks and follows approximately 500 HTLV-I seropositive patients. We recruited all consecutive patients presenting to routine clinic visits between 01/08/2004 and 16/03/2005. Sample size was determined by convenience. The inclusion criteria were: age > 18 years, HTLV-I seropositivity confirmed by Western Blot, and willingness to participate and sign an informed consent form. All patients underwent a thorough history and physical examination performed by an experienced neurologist as well as laboratory and image data, when deemed appropriate. The patients were divided into three groups according to the Expanded Disability Status Scale (EDSS) score [5]: 1) No neurologic involvement, when the EDSS was zero; 2) Incipient myelopathy; when 0.0 < EDSS < 3.0; and 3) HAM/TSP, when EDSS ≥ 3.0. Additionally, the diagnosis of HAM/TSP required a compatible clinical presentation, presence of HTLV-I antibodies in the CSF; and exclusion of alternative diseases that could be accounting for the neurologic disability [6]. All participants agreed to participate in the study and signed an informed consent form prior to the interview and urine collection. The research reported herein is in compliance with the Helsinki Declaration and was approved by the ethics committee of Fiocruz (number 029/2006).

### Study design

This is a cross sectional study. On the same day, all patients underwent a structured interview and provided a urine sample for culture and sensitivities (C&S). Patients were questioned about demographic data and presence of urinary symptoms suggestive of UTI, such as dysuria, urgency, frequency (defined as more than eight voids per day) and supra pubic discomfort. Information on the EDSS score was obtained from the patient's chart. The results of urodynamic studies were also obtained by chart review. This study did not have a protocol specified for therapy of UTI or further investigation and treatment of urinary symptoms. The clinical management of these patients was left up to the discretion of the clinic physician, who had access to all of the studies performed.

### Urine C&S

At the end of the clinic appointment, all patients provided a urine sample for C&S. After genital hygiene with mild soap, a mid-stream urine sample was collected on a sterile container and immediately sent to the microbiology laboratory. A calibrated loop (1/1000) was used to inoculate McConkey and sheep blood agar plates, which were then incubated at 37°C and inspected 24 hours later for the presence of bacterial growth. Plates that did not show any evidence of bacterial growth were re-incubated for an additional 24 hours. When bacterial growth was detected, a gram stain was performed. Gram-negative bacteria were identified using commercially available kits (Enterokits, PROBAC). Gram-positive bacteria were identified with the aid of biochemical studies and the coagulase test. For antimicrobial susceptibility testing, bacteria were inoculated onto Mueller-Hinton agar plates and incubated for 24 hours at 37°; sensitivities were determined by the disk diffusion method, using zone diameter standards for antibiotic susceptibility obtained from the National Committee of Clinical Laboratory Standards [7]. All urine C&S studies were performed at a single laboratory by the same technician (KS). A positive urine culture was defined as the growth of greater than 100.000 CFU/ml of a single bacterial species [8]. Contamination was defined as the growth of more than one species or the isolated growth of Lactobacillus, alpha-hemolytic Streptococcus, Gardnerella or Corynebacteria [9].

### Statistics

The data were summarized using mean ± standard deviation (SD) or percentages (%). For the variable 'age', we also split the population in intervals of two decades (starting at age 18, since this was the entry criteria) to provide further information about the age distribution of our patients. The Mann-Whitney U test was used to compare continuous variables (age) between two groups (HAM/TSP versus others and symptomatic versus asymptomatic). The Fisher's exact test was used to analyze the 2 × 2 table of urinary symptoms and urine culture results. Univariate and multivariate logistic regression analyses were performed to identify predictors of urinary symptomatology; the results of these analyses were reported as odds ratios (OR) and 95% confidence intervals (CI). All calculations were performed using SAS version 8.2 (Cary, NC). P values < 0.05 were considered statistically significant.

## Results

We prospectively interviewed 157 patients presenting to a routine clinic visit over a seven-month period. Mean (± SD) age was 48 ± 12 years and 55% were female (Table 1). The majority (52%) had no neurologic impairment, 27% had incipient myelopathy and 21% had HAM/TSP. When compared to the remainder of the population, HAM/TSP patients were significantly older (53 ± 14 versus 47 ± 12 years, P = 0.027), with a slight (57.6%) male predominance (data not shown).

**Table 1:** Characteristics of the population studied and urinary findings

Variable	n = 157
Age (mean ± SD), *years*	48 ± 12
18 – 40	24% (37)
40 – 60	64% (101)
60 – 80	11% (17)
> 80	1% (2)
Gender	
Male	45 % (71)
Female	55 % (86)
Marital status	
Single	31% (49)
Married	44% (69)
Other	25% (39)
EDSS score*	
0.0	52% (80)
1.0 – 3.0	27% (42)
≥ 3.0	21% (33)
Urinary Symptoms	
Present (at least one)	41% (64)
Absent	59% (93)
Urine Culture	
Positive	10% (16)
Negative	87% (136)
Mixed flora or < 100.000 CFU/ml	3% (5)

* Data available for 155 patients. Of the 33 patients with EDSS ≥ 3.0, most (n = 15) were in the 3.0 – 4.0.range. The remainders were distributed as follows: EDSS 4.0 – 6.0 = 6 patients; EDSS 6.0 – 8.0 = 8 patients; EDSS of 8.0 = 4 patients.

Upon direct questioning, 64 patients (41%) complained of at least one active urinary symptom suggestive of UTI (Table 1). Most of these patients (34/64 [53%]) reported only one urinary symptom; two, three and four symptoms were reported by 18, 04 and 08 patients, respectively. The most commonly reported symptom was supra-pubic discomfort (33%), followed by urgency (26%), frequency (25%) and dysuria (16%). There was no age difference between symptomatic and asymptomatic patients but most (71.9%) symptomatic patients were female (data not shown).

Bacterial growth was detected in the urine of 21 patients. In two cases, urine samples were judged contaminated due to polymicrobial growth; in three others, bacterial growth was less than 100.000 CFU/ml. Therefore, 16 patients (10%) were considered to have a positive urine culture (Table 1). The organisms isolated in these 16 cultures were: *E. coli *(09), *K. pneumoniae *(02), *Staphylococcus sp*. (02), *S. maltophilia *(01), *C. freundii *(01) and *P. mirabilis *(01). There was no statistically significant association between age and urine culture results but the vast majority of positive cultures (81.3%) occurred in women; only three men had positive urine cultures, two of them with HAM/TSP (EDSS scores of 3.0 and 8.0) and one with incipient myelopathy (EDSS score = 1.0) (data not shown).

**Figure 1:**
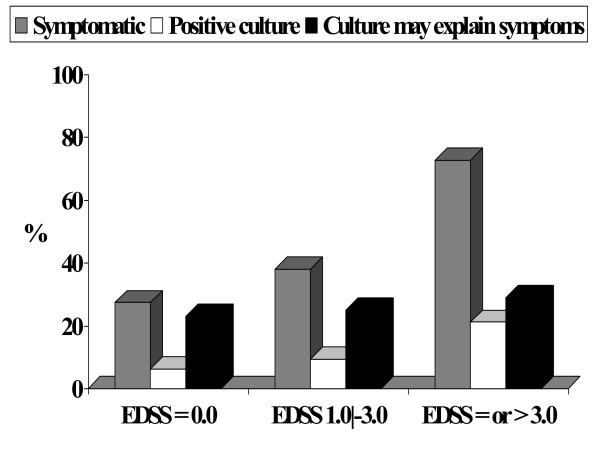
**Prevalence of urinary symptoms and positive urine culture stratified by the degree of neurologic impairment**. The gray bars represent the prevalence of urinary symptoms; the white bars, the prevalence of positive urine cultures; and the black bars, the percentage of urinary symptoms that may have been explained by a positive urine culture. Of the 80 patients without neurologic impairment (EDSS = 0.0), 22 (27.5%) had urinary symptoms and 05 (6.3%) had positive urine culture. Of the 42 patients with incipient myelopathy (EDSS 1.0–3.0), 16 (38.1%) had urinary symptoms and 04 (9.5%) had positive urine culture. Of the 33 patients with HAM/TSP (EDSS = 3.0), 24 (72.7%) had urinary symptoms and 07 (21.2%) had positive urine culture. A positive urine culture may explain the urinary symptomatology in 23% (5/22), 25% (4/16), and 29% (7/24) of patients without neurologic involvement, incipient myelopathy, and HAM/TSP, respectively.

Table 2 shows the correlation between urinary symptoms and urine culture results. Among asymptomatic patients, only four had significant bacteriuria; most asymptomatic patients (89/93 [96%]) had a negative urine culture. Of the 64 patients with urinary symptomatology, 12 patients (19%) had actual UTI confirmed by a positive urine culture. There was a correlation between urinary symptomatology and urine culture positivity as most positive cultures came from symptomatic patients (P = 0.006, Fisher's exact test). It should be noted, however, that urinary complaints could not be explained by presence of UTI in 81% of symptomatic patients. Fig. 1, shows the prevalence of urinary symptoms and positive urine culture stratified by the degree of neurologic impairment as determined by the EDSS score. Both prevalences increased directly in relation to EDSS scores but in most patients, the urinary complaints could not be explained by a positive culture.

**Table 2:** Correlation between urinary symptoms and urine culture results

	Urinary Symptoms		
		
Urine Culture	Absent	Present	Total (n)
Negative*	89 (96%)	52 (81%)	141
Positive	4 (4%)	12 (19%)	16

Total (n)	93 (100%)	64 (100%)	157

*We included five urine cultures that were either contaminated (n = 2) or had less than 100.00 CFU/ml (n = 3) among the Negative urine culture group.

To investigate the mechanism behind the urinary complaints in symptomatic individuals with negative urine cultures, we reviewed the results of urodynamic studies performed in 21 of these patients. Most of them (90.5%) had abnormal findings. The predominant abnormalities encountered in these studies were detrusor sphincter hyperreflexia (13/21 [61.9%]) and dyssynergia (4/21 [19%]); two patients (9.5%) were diagnosed with stress incontinence and two (9.5%) had completely normal urodynamic studies. Table 3 shows the urodynamic findings distributed according to EDSS scores. Voiding dysfunction was not limited to patients with HAM/TSP as it was found in all patients without neurologic involvement and in five out of six patients with incipient myelopathy.

**Table 3:** Urodynamic Findings Distributed According to EDSS Scores in 21 Patients with Urinary Symptomatology and Negative Urine Cultures

	EDSS Score			
		
Urodynamic Findings	0.0	1.0 – 3.0	≥ 3.0	Total (n)
Normal	0	1	1	2
*DS Hyperreflexia	5	3	5	13
*DS Dyssynergia	0	1	3	4
Stress Incontinence	0	1	1	2

Total (n)	5	6	10	21

*DS – detrusor sphincter

To examine the association between several covariates and the presence of urinary symptoms, we performed univariate and multivariate logistic regression analyses (Table 4). On the univariate analysis, female gender, an abnormal EDSS score, and a positive urine culture were significantly associated with increased odds of urinary symptoms. On the multivariate analysis, the only independent risk factors for urinary symptomatology were female gender and an abnormal EDSS score, with the latter showing the most significant association (OR 9.87, 95% CI 3.465 to 28.116, P < 0.0001).

**Table 4:** Predictors of urinary symptomatology in HTLV-I-infected individuals

	Univariate Logistic Regression			Multivariate Logistic Regression		
	
Variables	OR	95% CI	P value	OR	95% CI	P value
Age	1.01	0.98 to 1.03	0.5975	1.00	0.97 to 1.03	0.7535
Gender (fem. vs. male)	3.39	1.71 to 6.70	0.0003	5.05	2.13 to 11.96	0.0002
EDSS (≥ 1.0 vs. 0.0)	5.89	2.50 to 13.88	< 0.0001	9.87	3.47 to 28.12	< 0.0001
Urine culture (pos. vs. neg.)	5.14	1.57 to 16.75	0.0033	2.870	0.78 to 10.61	0.1141

Footnote: N = 155 (2 missing data for EDSS). Age was used as a continuous variable; fem. = female; pos. = positive; neg. = negative; OR = odds ratio; CI = confidence intervals.

## Discussion

In the current study, we have interviewed and performed urine cultures on 157 consecutive HTLV-I-infected individuals presenting to a routine clinic visit to determine the relation between active urinary symptomatology and the presence of UTI. Several findings are noteworthy.

We encountered 64 patients complaining of at least one urinary symptom; in 12 of these patients, the diagnosis of UTI was subsequently confirmed by a positive urine culture. However, the vast majority (n = 52) of patients with active urinary symptoms suggestive of UTI had negative urine cultures. Upon further investigation of a subgroup of these patients with urodynamic studies, we detected findings suggestive of neurogenic bladder in 81% (17/21) of them. We found thirteen cases of detrusor sphincter hyperreflexia and four cases of detrusor sphincter dyssynergia. These are the most frequently encountered types of voiding dysfunction among patients with neurogenic involvement of the bladder by HTLV-I [10-18]. In our study, these abnormalities on urodynamic evaluation were found even in patients with normal EDSS scores (Table 3), perhaps suggesting that this scale might not have adequate sensitivity to detect very early urologic abnormalities in HTLV-I patients. It should be noted that since the selection of patients for urodynamic evaluation in this study was done on clinical grounds, it is possible that the high incidence of neurogenic bladder encountered in these 21 patients represent a selection bias.

In a multivariate logistic regression analysis an abnormal EDSS score was the strongest predictor of urinary symptomatology: individuals with abnormal EDSS scores (= 1.0) had almost 10 times greater odds of having urinary symptoms than individuals with EDSS scores of zero. Nevertheless, we still found a significant prevalence of urinary complaints (27.5%) among individuals with EDSS scores of zero, adding to our reservation regarding the sensitivity of this scale in this setting. Female gender was also an independent predictor of urinary symptoms. A positive urine culture was associated with urinary symptomatology on univariate analysis but lost statistical significance on the multivariate model probably due to an interaction with the variables gender and EDSS; this is because most of the positive urine cultures occurred in females (13/16 [81%]) and in patients with abnormal EDSS (11/16 [69%]).

Murphy et al. have described an increased prevalence and incidence of UTI among HTLV-I seropositive individuals compared to a control group of seronegative blood donors [1-3]. In their studies, however, the diagnosis of UTI relied solely on patient's recollection, which may have led to a recall bias in the HTLV-I seropositive group. More importantly, even though patients were told to report only episodes of UTI that were identified and treated by a physician, this does not necessarily guarantee an accurate diagnosis. Patients, especially young females, are often treated empirically for UTI based on their symptoms, without confirmation by a urine culture [19]. Therefore, it is plausible that in the studies by Murphy et al., HTLV-I-infected patients with urinary symptoms due to neurogenic bladder may have been misdiagnosed as having UTI.

There are limitations to our study. Since UTI is an acute condition that resolves with treatment, we recognize that measuring its punctual prevalence during pre-scheduled, routine visits to an HTLV-I ambulatory clinic clearly underestimates the burden of UTI in this population. However, the main objective of our study was not to detect the prevalence of UTI but rather the prevalence of active urinary symptoms and identify what percentage of these could be explained by UTI; therefore, we feel that our study design and location were adequate to address this issue. Another limitation is the lack of a seronegative control group. Without the seronegative group, our results cannot dispute the assertion of Murphy et al. that UTI is more common in HTLV-I seropositive individuals. Nevertheless, the current study indicates that defining UTI in this population based on data obtained from an interview is inadequate and may lead to over diagnosis by detecting a large proportion of patients with neurogenic bladder that do not have infection.

## Conclusion

Our study revealed a high prevalence of urinary symptoms among HTLV-I-infected individuals but most of these patients did not have UTI. Urodynamic studies performed in patients with active urinary symptomatology and negative urine cultures revealed findings consistent with neurogenic involvement of the bladder by HTLV-I. Additionally, an abnormal EDSS score was a strong predictor of urinary symptomatology. We conclude that neurogenic bladder might be an early sign of HAM/TSP and the most likely cause of urinary symptomatology in HTLV-I-infected individuals presenting to a routine clinic visit. Our data implies that HLTV-I infected patients with urinary symptomatology should not be empirically treated for UTI but rather undergo urine culture; if a UTI is excluded, further investigation with urodynamic studies should be considered.

## Competing interests

The author(s) declare that they have no competing interests.

## Authors' contributions

PNR participated in the conception and design of the study, analyzed and interpreted the data and wrote the manuscript. APR and JFS performed the medical interviews. ALM performed the neurological examination. NC performed the urodynamic studies. KS performed the urine cultures. HR and EMC conceived of the study, and participated in its design and coordination. *All authors performed a critical review of the initial draft for important intellectual content and approved the final manuscript (*HR passed away before he could approve the final manuscript).

## Pre-publication history

The pre-publication history for this paper can be accessed here:


